# Protocol for EHS Rapid Guideline: Systematic Review, Meta-Analysis, GRADE Assessment, and European Recommendations on Parastomal Hernia Prevention

**DOI:** 10.3389/jaws.2022.10509

**Published:** 2022-05-20

**Authors:** Stavros A. Antoniou, Cesare Stabilini, Ourania Koutsiouroumpa, Dimitrios Mavridis, Filip Muysoms

**Affiliations:** ^1^ Surgical Department, Mediterranean Hospital of Cyprus, Limassol, Cyprus; ^2^ Medical School, European University of Cyprus, Nicosia, Cyprus; ^3^ Department of Surgery, University of Genoa, Genoa, Italy; ^4^ Department of Primary Education, School of Education, University of Ioannina, Ioannina, Greece; ^5^ Faculté de Médecine, Université Paris Descartes, Paris, France; ^6^ Department of Surgery, Maria Middelares Hospital, Ghent, Belgium

**Keywords:** parastomal hernia, mesh, prophylactic, prevention, rapid guideline, GRADE, AGREE-S, EHS

## Abstract

**Background:** Parastomal hernia presents frequently after construction of a permanent end colostomy. Previous guidelines recommend using a prophylactic mesh for hernia prevention. Randomized controlled trials (RCTs) published hereafter demonstrate conflicting outcomes.

**Methods and Analysis:** A rapid guideline will be developed and reported in accordance with GRADE, GIN and AGREE-S standards. The steering group will consist of general and colorectal surgeons, members of the EHS Scientific Advisory Board with expertise and experience in guideline development, advanced medical statistics and evidence synthesis, and a certified guideline methodologist. The guideline panel will consist of three general surgeons, three colorectal surgeons, two stoma care nurses, and two patient representatives. A single question will address the safety and efficacy of the use of a prophylactic mesh in patients with a permanent end colostomy, and sensitivity analyses will focus on the use of non-absorbable versus absorbable meshes, and on different anatomical spaces for mesh placement. A systematic review will be conducted and evidence synthesis will be performed by statisticians independently. The results of evidence synthesis will be summarized in summary of findings tables. Recommendation(s) will be finalized through Delphi process of the guideline panel within an evidence-to-decision framework.

**Ethics and Dissemination:** The funding body will not be involved in the development of this guideline. Conflicts of interest, if any, will be addressed by re-assigning functions or replacing participants with direct conflicts, according to Guidelines International Network recommendations.

## Introduction

### Background

Parastomal hernia is encountered frequently after permanent end colostomy. Previous systematic reviews suggest that the absolute risk of parastomal hernia is 30% by 12 months and 50% beyond 2 years (1). Using a prophylactic mesh, synthetic or absorbable, has been suggested to reduce the risk of parastomal hernia without increasing perioperative and longer-term stoma-related complications (2,3). Previous guidelines of the European Hernia Society provided a strong recommendation for the use of a prophylactic mesh when constructing an end colostomy (1). More recent data from randomized controlled trials (RCTs) provided conflicting evidence, giving rise to a debate over the optimum management of patients who are planned to have an end colostomy (4,5).

### Objective

In view of the new evidence and under consideration of the evolvement in the methodology of guideline development, the objective of this rapid guideline is to provide transparently developed, reliable, and evidence-informed update recommendation(s) on the use of prophylactic mesh for the prevention of parastomal hernia in patients who will have an end colostomy.

## Methods

The present protocol adheres to AGREE-S and PRISMA-P reporting standards (6,7). It will be made available on the EHS website for access by healthcare professionals, and EHS members will be invited through various channels (social media, email newsletter) to comment on the content. Relevant comments will be considered by the steering group.

### Steering Group

The steering group consists of general and colorectal surgeons with specific interest in hernia surgery, members of the EHS Scientific Advisory Board, and experts in guideline development and evidence synthesis.

### Guideline Methodologist

The first author is a certified guideline methodologist (INGUIDE certificate number 2021-L2-V1-00001), has participated in the development of more than 15 clinical practice guidelines, has vast experience in evidence synthesis, and will serve as a guideline methodologist.

### Evidence Outreach Team

An evidence outreach team will consist of at least two healthcare professionals with experience in evidence outreach. They will be free of direct and indirect conflicts, and they will act independently from the steering group, they will however consult the guideline methodologist and the guideline panel, as per GRADE standards.

### Guideline Panel and External Advisors

The guideline panel will consist of three colorectal surgeons, three general surgeons with specific interest in hernia surgery, two stoma care nurses and two patient representatives. The composition of panel members aims for representation of both genders, different parts of Europe, and academic and non-academic practice. We will ask for input from surgeons who have published RCTs and/or meta-analyses on this topic, however they will not have voting privileges on the direction and the strength of the recommendation(s) within the evidence-to-decision framework, due to indirect conflicts, as per Guidelines International Network guidelines (8). The guideline panel’s and external advisors’ contribution will be acknowledged by authorship in the resulting journal publication (9).

### PICO Question

Should prophylactic mesh versus no prophylactic mesh be used in patients who undergo construction of a permanent end colostomy?

Sensitivity/subgroup analysis will address the use of absorbable versus non-absorbable meshes and different anatomic spaces of mesh placement.

### Guideline Development Methodology

The guideline development process will adhere to AGREE-S and GRADE guideline development standards, and methodology parameters of rapid recommendations (6,10) and guideline development processes summarized by the Guidelines International Network (11). The guideline panel will be asked to comment on the PICO questions and subgroup analyses, and they will be surveyed to nominate important and critical outcomes, and to define minimal important differences.

The literature search strategy will be developed by a member of the steering group with experience in outreach, knowledge, and evidence search, and it will be built upon the previous systematic review from January 2016 to the present (Supplementary Appendix) (1). The Healthcare Databases Advanced Search (HDAS) interface developed by the National Institute for Health and Care Excellence (NICE) will be used to interrogate Medical Literature Analysis and Retrieval System Online (MEDLINE). The grey literature will be searched through OpenGrey (Exalead). Relevant terms will be selected to identify eligible reports. Thesaurus headings, search operators, and search limits in each of the above databases will be adapted accordingly.

Risk of bias of eligible studies will be assessed using RoB 2 for randomized trials (12). Study selection will be performed by the evidence outreach team; risk of bias assessment and data extraction will be performed by one member of the evidence outreach team and independently cross-checked by the guideline methodologist. Statistical analyses will be performed by biostatisticians using the methodology reported below.

GRADE evidence tables will be developed by the panel with methodological advice from the methodologist in a face-to-face consensus meeting of the guideline development group. Draft recommendations will be formulated and strength of recommendations will be defined. The recommendations may be refined and their strength may be revised in line with the results of an online Delphi process of panel members. Comments by the Delphi panel must be in accordance with the GRADE methodology in order to be considered. Formulation of recommendations will be informed by GRADE and AGREE-REX (13,14).

### Time-to-Event Data Meta-Analysis

For trials including time-to-event outcomes (e.g., elapsed time before a hernia occurs), we will extract the hazard ratio and the standard error of the logarithm of the hazard ratio. If instead of the standard error, the corresponding 95% confidence interval or *p*-value is reported, we can used them to estimate the standard error. These are standard outputs from a Cox proportional hazards model. If these data are not given, we can approximately estimate the hazard ratio using statistics estimated from a long-rank analysis. If some studies give the hazard ratio but other give risk ratios (or the number of events and sample size in each group), we will combine them in a sensitivity analysis.

### Pairwise Meta-Analysis

Mantel-Haenszel pooled odds ratios (or the risk difference in the presence of zero events in at least one group, in one or more studies) will be calculated for binary variables and the standardized mean difference for continuous variables, with corresponding 95% confidence intervals. Where means and *p*-values will be given, the standard error and the standard deviation will be estimated by calculating the standard error and t-value using the given degrees of freedom. The standard error and the standard deviation will be obtained from confidence intervals by using the formula suggested by the Cochrane Collaboration (15).

Conceptual heterogeneity related to the PICO parameters and the study design will be assessed, and statistical heterogeneity will be explored using the *I* ^*2*^ statistic. We will conduct a random effects meta-analysis to estimate the pooled effect and its 95% confidence interval. We will also compute prediction intervals. As a sensitivity analysis, we will conduct a fixed effect meta-analysis. We will explore for small-study effects using funnel plots and statistical tests (e.g., Egger’s test) (16). Statistical analyses will be performed using the meta library in R (17,18).

### Target Users

This guideline is intended to be used by general and colorectal surgeons, multidisciplinary team members, stoma care nurses, hospital administrators, policy makers, and patients. The guideline publication will contain a short abstract in plain language to be used by patients.

### Publication and Dissemination Strategy

As a EHS-sponsored project, this guideline will be submitted for publication in the Journal of Abdominal Wall Surgery, official journal of the EHS.

### Feedback

The steering group will consider constructive feedback received during the conduct of the project via various routes and sources such as letters to the editor and social media. Such feedback will be taken into account in the guideline development process or a future update of the guideline.

### Monitoring, Update and Future Steps

Use of the guideline by EHS members will be monitored through an online survey 2 years after publication. The timing of the update of the guideline will be decided by the steering group on the basis of new research data on this topic.

## Discussion

### Implications for Practice and Research

Stringent criteria defined by GRADE and AGREE-S will be applied to collate, appraise and analyze the available evidence. The guideline is expected to inform decision making, and guide clinical practice and health policy. Guidance will be provided on direction and implications for future research in light of identified evidence gaps.

### Strengths and Limitations

The strengths and limitations of rapid guidelines have been previously reported (19-21). The merits of rapid guidelines, including trustworthiness, credibility, and time efficiency have to outweigh the shortcomings, such as the narrow scope and possible missing of resources due to the rapid review process.

### Research Ethics

EHS, as the funder, will not be involved in the development of this guideline. Research Ethics Committee approval is not necessary as this project does not involve any identifiable patient data. Conflicts of interest statements will be collected by all guideline panel members before and upon completion of the project. Panel members with direct conflicts will be replaced and participants with indirect conflicts will be re-assigned functions, according to Guidelines International Network recommendations (18).

## Conclusion

This rapid guideline will address the use of prophylactic mesh for parastomal hernia prevention in patients who will have an end colostomy. It is expected to provide useful, evidence-based and stakeholder-informed information of the most appropriate management in this context.
