# Supplementary material for: Protocol for EHS Rapid Guideline: Systematic Review, Meta-Analysis, GRADE Assessment, and European Recommendations on Parastomal Hernia Prevention
Source: J Abdom Wall Surg. 2022 May 20;1:10509. doi: 10.3389/jaws.2022.10509 (PMC10831635; doi:10.3389/jaws.2022.10509)
Supplement: Supplementary file 1 [file DataSheet1.docx]

**Appendix: Search strategy**

*MEDLINE*

1. surgical mesh [mh]

2. mesh [tiab, tw]

3. prevention and control [sh]

4. prophyla* [tiab, tw]

5. prevent* [tiab, tw]

6. hernia, abdominal [mh]

7. hernia, incisional [mh]

8. parastoma* hernia [tiab, tw]

9. para-stoma* hernia [tiab, tw]

10. stoma [tiab, tw]

11. *ostomy [tiab, tw]

12. ostomy [mh]

13. ostomy [tiab, tw]

14. ileal conduit [tiab, tw]

15. Bricker [tiab, tw]

16. #1 OR #2

17. #3 OR #4 OR #5

18. #6 OR #7

19. #8 OR #9 OR #10 OR #11 OR #12 OR #13 OR #14 OR #15

20. #16 AND #17 AND #18 AND 19

Search string: (surgical mesh [mh] OR mesh [tiab] OR mesh [tw]) AND ((prevention and control [sh]) OR prophyla* [tiab] OR prophyla* [tw] OR prevent* [tiab] OR prevent* [tw]) AND (hernia [mh] OR hernia [tiab] OR hernia [tw]) AND (stoma [tiab] OR stoma [tw] OR *ostomy [tiab] OR *ostomy [tw] OR ostomy [mh] OR ostomy [tiab] OR ostomy [tw] OR (ileal conduit [tiab]) OR (ileal conduit [tw]) OR Bricker [tiab] OR Bricker [tw])

*OpenGrey*

Search string: mesh AND (prophyla* OR prevent*) AND hernia AND (stoma OR *ostomy OR (ileal conduit) OR Bricker)
